# Clinical value and molecular mechanism of AQGPs in different tumors

**DOI:** 10.1007/s12032-022-01766-0

**Published:** 2022-08-16

**Authors:** Rui Wang, Xuejian Wang, Jun Zhao, Jiacheng Jin, Weiwei Fan, Xinqing Zhu, Qiwei Chen, Baochang Zhang, Lan Lan, Kexin Qu, Liang Zhu, Jianbo Wang

**Affiliations:** 1 Department of Urology, The First Affiliated Hospital of Dalian Medical University, Dalian, 116021 China; 2 Department of Urology, The Third People’s Hospital of Chengdu, Chengdu, 610014 China; 3 College of Basic Medical Science, Dalian Medical University, Dalian, 116044 China

**Keywords:** Aquaglyceroporin, Cancer, Expression pattern, Regulator, Signaling pathway

## Abstract

Aquaglyceroporins (AQGPs), including AQP3, AQP7, AQP9, and AQP10, are transmembrane channels that allow small solutes across biological membranes, such as water, glycerol, H_2_O_2_, and so on. Increasing evidence suggests that they play critical roles in cancer. Overexpression or knockdown of AQGPs can promote or inhibit cancer cell proliferation, migration, invasion, apoptosis, epithelial–mesenchymal transition and metastasis, and the expression levels of AQGPs are closely linked to the prognosis of cancer patients. Here, we provide a comprehensive and detailed review to discuss the expression patterns of AQGPs in different cancers as well as the relationship between the expression patterns and prognosis. Then, we elaborate the relevance between AQGPs and malignant behaviors in cancer as well as the latent upstream regulators and downstream targets or signaling pathways of AQGPs. Finally, we summarize the potential clinical value in cancer treatment. This review will provide us with new ideas and thoughts for subsequent cancer therapy specifically targeting AQGPs.

## Introduction

Water is the source of life. Water entering and leaving cells are a basic process of metabolism. In the very beginning, simple diffusion was considered to be the major route of water, but membrane water permeability shown by some epithelia was too high to just be explained by simple diffusion, which inspired explorations on the existence of water-specific channels [1]. Until the historic discovery of a novel 28 kDa integral membrane protein (CHIP28) in human erythrocytes [2, 3], people gradually uncovered the veils of water channel proteins. Then, CHIP28 was named aquaporin 1(AQP-1) after its water transport functions were proven by Peter Agre et al. in Xenopus oocytes [4–7].

Aquaporins exert a profound influence on the regulation of water homeostasis by providing selective pores for the rapid movement of water across diverse cell membranes and regulating cell volume [8]. To date, 13 aquaporins have been found in mammals. Among them, orthodox aquaporins are permeable to water. However, aquaglyceroporins (AQGPs), another subclass of aquaporins, including AQP3, AQP7, AQP9, and AQP10, are capable of facilitating the transport of some small molecules across the membrane, especially glycerin and urea, in addition to water. They were noted to be genetically close to the known E.coli glycerol transport protein GlpF [9], and thus, they were also classified as “the GlpF group.”

Cancer is a threat to human health. Lipid metabolism is receiving much attention in cancer research today. Cancer cells rely on abnormal lipid metabolism to proliferate, metastasize, and adapt to the tumor microenvironment (TME) [10]. Moreover, aberrant uptake, storage, synthesis, and utilization of lipids have been detected in many cancers, and directly exacerbated tumorigenicity and malignancy [11]. Emerging evidence also shows that the functions of immune cells in the TME are closely related to abnormal lipid metabolism [12]. Aquaglyceroporins, as channels for glycerol, determine glycerol trafficking in and out of cells and subsequent lipid metabolism. Research has demonstrated that silencing AQP3 contributes to proliferation impairment and apoptosis via decreased glycerol uptake and lipid synthesis in gastric cancer cells [13, 14]. Hara-Chikuma and Verkman found that glycerol permeability via AQP3 is required for epidermal cell proliferation and tumorigenesis, as cellular glycerol is a key determinant of cellular ATP energy [15]; also, AQP3/PLD2 signaling module may be involved in the process of converting glycerol to phosphatidylglycerol in squamous cell carcinoma and basal cell carcinoma [16]. In mouse breast cancer models, lipid accumulation in Aqp7 KD tumors was detectable by Oil Red O staining [17]. Moreover, AQP9 participates in hepatic glycerol metabolism reprogramming in early rat liver cancer [18]. Therefore, in this review, we focus on aquaglyceroporins, which are not only channels for glycerin and water transportation, but also important biomarkers for predicting tumor prognosis and affecting malignant behaviors.

To date, no systematic review has further explored the relationship between aquaglyceroporins and cancer. In this review, we analyze how AQGPs affect the malignant behaviors of cancer by investigating the expression patterns of AQGPs and their relationship with cancer prognosis in hope of some new ideas beneficial to cancer treatment.

## Structure

Members of the AQGPs show similar topology, including six nonpolar membrane-spanning domains of sufficient length, five connected loops consisting of three extracellular loops (A, C, E), and two intracellular loops (B, D), cytoplasmic-facing NH2 and COOH termini, and highly conserved motifs covering two tandem repeat Asp-Pro-Ala sequences (NPA box) located in loops B and E, respectively, one “AEFL” and one “HW[V/I][F/Y]WXGP” sequence[19–21]. The three-dimensional “hourglass model” is composed of homotetramers, and each monomer of the homotetramers has a functional water channel [22].

**Fig. 1:**
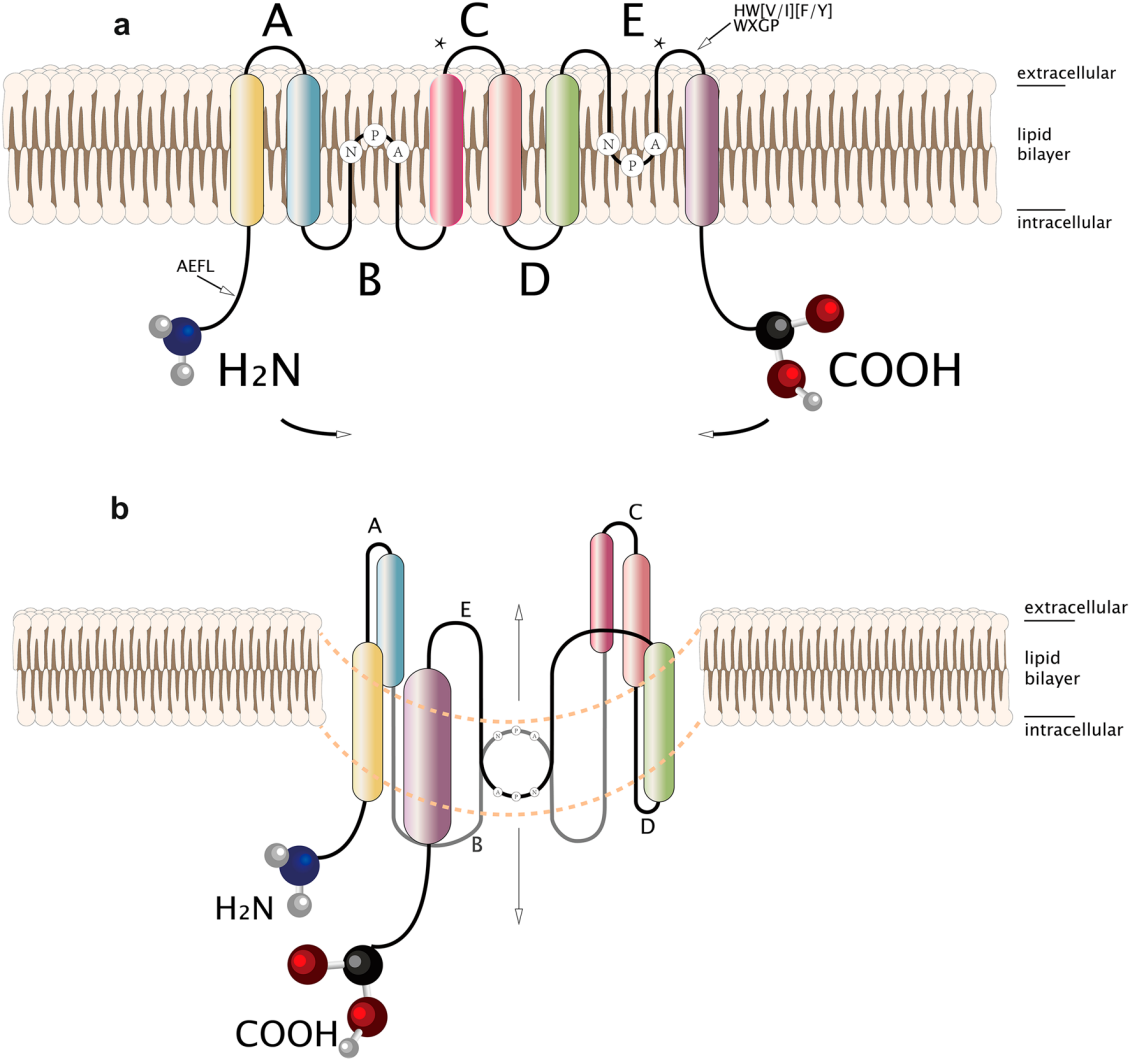
**a** Locations of NPA boxes, membrane-spanning domains, cytoplasmic-facing terminus are shown, and two additional peptide spans are denoted by asterisks; **b** Hourglass model for aquaglyceroporin membrane topology

Surprisingly, the presence of two additional peptide spans, one in loop C and the other in loop E after the second NPA motif, was observed in all aquaglyceroporins but not in orthodox aquaporins[9, 19, 23]. Although the structural explanation for the functional difference between aquaporins and aquaglyceroporins has not reached a consensus today, these distinctive domains may be the key [9, 19] (See Fig. 1)

## Expression patterns and relationship with cancer prognosis

### AQP3

AQP3 was the first aquaglyceroporin known and studied in humans and is expressed in a variety of tissues, including the renal collecting duct [24], respiratory epithelium [25], breast [26], stomach [27], and prostate [28]. Recently, an increasing number of researchers have pointed out that AQP3 is inclined to be of considerable importance in cancer development, which indicates that it may serve as a biomarker of cancer prognosis.

A recent study provided insight into the possible etiological theory that positive AQP3 expression was related to lymph node metastasis, invasion, and high TNM stage in patients with pancreatic ductal adenocarcinoma (PDAC) [29]. In addition, AQP3 expression was reinforced in later and more aggressive stages of PDAC [30]. Another study suggested that AQP3, regulated by estrogen, might be adopted as a diagnostic biomarker for the early detection of ovarian cancer [31]. Furthermore, enhanced expression of AQP3 was also correlated with lymph node metastasis in patients with colon and gastric cancer [32, 33]. In addition, a Chinese research team highlighted that preoperative serum AQP3 levels were significantly elevated in patients diagnosed with colon cancer, demonstrating its clinical value for the early screening of colon cancer [34]. Protein or mRNA expression levels of AQP3 are related to the TNM stage, lymph node status, relapse, metastasis, and some other clinical indicators, which ultimately contribute to cancer outcomes. See Table 1 for the relationship between AQP3 expression levels and cancer prognosis in detail.

**Table 1:** Relationship between AQP3 expression levels and cancer prognosis

Cancer types	Expression types	Expression and prognosis
Gastric carcinoma [35, 36]	mRNA	Higher expression is correlated with better OS
Pancreatic ductal adenocarcinoma [29]	protein	Higher expression is correlated with worse OS
Esophageal squamous cell carcinoma [37]	protein	Co-expression of AQP3 and AQP5 shows worse OS + DFS
Triple-negative breast cancer [38]	protein	Higher AQP3 and AQP5 expression shows worse OS + DFS
Breast cancer [39]	mRNA	Higher expression is correlated with worse RFS
HER2-positve early breast cancer [40]	protein	40.3% positive expression shows worse DFS
HER2-positive early breast cancer [41]	mRNA	Positive expression shows worse RFS
Hepatocellular carcinoma [42]	protein	Higher expression is correlated with worse OS + DFS
Endometrioid carcinoma [43]	protein	Positive expression shows better OS + PFS
Urothelial carcinoma [44–47]	protein	High expression in CIS: usually poor prognosis MIBC: higher expression means better PFS
Ovarian cancer[48]	mRNA	Higher expression is correlated with better OS

*OS* overall survival, *DFS* disease-free survival, *RFS* relapse-free survival, *PFS* progression-free survival, *HER2* human epidermal growth factor receptor 2, *CIS* carcinoma in situ, *MIBC* muscle-invasive bladder cancer

As shown in Table 1, AQP3 is also expressed in many cancer tissues and cells, but its expression patterns differ from those of cancers. Given its relationship with cancer prognosis, we delve into its expression patterns in different cancers hoping for some new discoveries. Table 2 shows the expression levels of AQP3 in cancer tissues or cells and corresponding normal tissues or cells.

**Table 2:** Expression levels of AQP3 in cancer tissues or cells and corresponding normal tissues or cells

Cancer types	Tissues/cell lines	Methods	Expression of tumors (T) and normal tissues or cells (N)
GC [33]	tissues	RT-PCR, IF, WB	T > N
GC [49]	tissues	WB	T > N
GC [50, 51]	tissues	IHC	T > N
GC [14]	tissues	RT-PCR	T > N
GC [36]	tissues	TCGA database	T < N
HCC [42]	tissues	IHC	T > N
HCC [52]	tissues	qRT-PCR, WB, IHC	T > N
HCC [53]	tissues	qRT-PCR, WB	T > N
HCC [54]	tissues	qRT-PCR, WB, IHC	T > N
Breast cancer [38]	tissues	IHC	T > N
Breast cancer [39]	tissues	Oncomine database	T < N
Breast cancer [55]	tissues	qRT-PCR	T > N
PDAC [29]	tissues	WB, IHC	T > N
PDAC [30]	tissues	IHC	T > N
SCC [16]	tissues (skin)	IHC	T > N
SCC [56]	tissues (esophagus, oral)	IHC	T > N
SCC [57]	tissues (oral)	IHC	T > N
SCC [37]	tissues (esophagus)	IHC	T > N
NSCLC [58]	tissues, cell lines	qRT-PCR, WB	T > N
Colorectal carcinoma [59]	tissues	IHC	T > N
Prostate cancer [60]	cell lines	qRT-PCR, WB	T > N
Osteosarcoma [61]	tissues, cell lines	qRT-PCR	T > N
Ovarian carcinoma(hen) [31]	tissues	qRT-PCR, IHC	T > N
Nonmelanoma skin cancer [62]	tissues (skin)	IHC	BCC < SCC < N

*GC* gastric carcinoma,* HCC* hepatocellular carcinoma, *PDAC* pancreatic ductal adenocarcinoma, *SCC* squamous cell carcinoma, *NSCLC* non-small cell lung carcinoma, *BCC* basal cell carcinoma

*RT-PCR* reverse transcription *PCR, IF* immunofluorescence, *WB* western blot, *IHC* immunohistochemistry, *qRT-PCR* quantitative real-time PCR

From Table 2, the expression level of AQP3 in most cancers is higher than that in the corresponding normal tissues or cells, particularly at the protein level, except for nonmelanoma skin cancer. From Table 1, at the protein level, overexpression of AQP3 or AQP3-positive often contributes to a worse prognosis except for endometrioid carcinoma and MIBC, indicating that AQP3 frequently acts as a villain in cancer.

The expression patterns of AQP3 in thyroid cancer, breast cancer, and prostate cancer are exceptional. In the thyroid, AQP3 expression was positive only in parafollicular cells (C cells). Nevertheless, in thyroid cancer, AQP3 mRNA and protein were only identified in medullary thyroid cancer derived from C cells [63], which might be interpreted as stimulation by hormones secreted by C cells such as calcitonin. In breast cancer, the highest level of expression of AQP3 was observed in endocrine-sensitive (YS1.2) breast cancer cells, followed by endocrine -resistant (pII) breast cancer cells, and the weakest expression was found in normal breast epithelial cells (MCF10A) [64], implicating that estrogen might act as an upstream regulator of AQP3. For prostate cancer (PC), our team demonstrated that AQP3 was primarily expressed in the membranes in the normal prostate epithelia, but in prostate cancer epithelia, AQP3 was often located in the cytoplasm [28]. Insang Hwang et al. achieved similar results [65]. It is worth noting that another study showed that AQP3 was expressed in the membrane and cytoplasm of LNCaP cells, an androgen-dependent cell line, and mainly in the cytoplasm of PC3 and Du145 cells, which are androgen-independent [66]. In summary, we observed an interesting phenomenon. In normal prostate epithelia, AQP3 is mainly found in the membrane. However, as the disease progresses to androgen-dependent PC, AQP3 often lies in the membrane and cytoplasm. When the disease progresses to the castration resistance stage, it mainly appears in the cytoplasm. Since the key to the pathological progression of prostate cancer is androgen, we hypothesize that androgen may also be responsible for the tendency of AQP3 to translocate from the cell membrane to the cytoplasm as prostate cancer progresses. Unfortunately, little work has been performed on our conjecture thus far. It seems reasonable that AQP3 can be regulated by hormones, including androgen, estrogen, and calcitonin. However, whether other hormones in the body have an influence and how they work is an issue that urgently needs to be verified. All of the above results show that AQP3, as a functional protein, is important for forecasting the prognosis of some cancers and indirectly indicates its feasibility as a therapeutic target.

### AQP7 and AQP10

Studies have shown that in addition to its rich expression in fatty cells, AQP7 is also expressed in other tissues, such as kidney, testis, heart, muscle, pancreas, and small intestines, to varying degrees [27, 67–70], and its main function is transporting water and glycerol.

AQP7 has different expression levels between tumors and corresponding normal tissues, implying that it may affect the prognosis of cancer. As shown in Table 3, we determined that the expression level of AQP7 mRNA in cancer tissues was often lower than that in the corresponding normal tissues, but protein-level evidence still needs to be discovered. Research suggests that the protein expression level of AQP7 in HCC and ovarian carcinoma tissues is significantly different from that in normal tissues, but its clinical significance remains to be explored.

**Table 3:** AQP7 expression levels in cancer tissues or cells and corresponding normal tissues or cells and relationship between AQP7 expression levels and cancer prognosis

Cancer types	Tissues/cell lines	Expression types	Expression of tumors (T) and normal tissues or cells (N)	Expression and prognosis
Low-grade glioma [48]	TCGA database	mRNA	T < N	Lower expression means better OS
ccRCC [71]	TCGA database	mRNA	T < N	Lower expression means worse OS
Breast cancer[39]	Oncomine database	mRNA	T < N	Lower expression means worse OS (in Grade 1)
Breast cancer [17]	TCGA database	mRNA	——	Lower expression means better OS
Breast cance [17] (mouse)	qRT-PCR IHC	mRNA, protein	T < N	——
PDAC [72]	GEO database	mRNA	T < N	Lower expression means worse OS
HCC [73]	qRT-PCR WB IHC	mRNA, protein	T < N	——
Ovarian carcinoma [74]	WB	protein	T > N	——

*ccRCC* clear cell renal cell carcinoma

AQP10, permeable to water, glycerol, and urea, is expressed in the digestive tract [75–77]. However, present studies have not been particularly informative about its role in cancer. Despite being a part of the AQGP family, its function remains unknown. Until now, AQP10 mRNA has been found in several cancers, such as breast cancer and ovarian cancer [78, 79]. A study of ovarian cancer demonstrated that higher AQP10 mRNA expression meant a better OS [79], and Lizhe Zhu et al. found that increased AQP10 mRNA expression in breast cancer was associated with better RFS [39]. In contrast, another study obtained the opposite result that AQP10 mRNA expression was relevant to poor OS [35].

The relationship between the expression levels of AQP7 and AQP10 and the prognosis of cancer remains ambiguous because there have been only a few attempts to examine AQP7 and AQP10 in cancer, and existing research was limited to the mRNA level. The identification and location of AQP7 and AQP10 at the protein level may be of considerable significance.

### AQP9

AQP9 is widely distributed in the body, including the nerve, digestive, and reproductive systems [80–84]. Although its molecular structure and water permeability are closely analogous to those of other aquaglyceroporins, relatively little is known about its specific physiological functions. AQP7 of adipocytes transports the glycerol produced by fat mobilization to the blood. After the blood enters the liver through the portal vein, AQP9 expressed in the liver facilitates the uptake of glycerol, and then, glucose is produced by gluconeogenesis [85]. In addition, it also plays a role in tumorigenesis, progression, and even metastasis. Similarly, we explored its expression levels in different cancer tissues or cells and corresponding normal tissues or cells. According to Table 4, the expression levels of AQP9 in hepatocellular carcinoma, lung cancer, and laryngeal cancer are lower than those in corresponding normal tissues, but the opposite result is observed in other cancers. Then, we compared the expression levels of AQP9 with cancer prognosis (Table 5). It is reasonable that AQP9 promotes cancer except for hepatocellular carcinoma.

**Table 4:** Expression levels of AQP9 in different tumor tissues or cells and corresponding normal tissues or cells

Cancer types	Tissues/cell lines	Methods	Expression of tumors (T) and normal tissues or cells (N)
HCC [86, 87]	tissues, cell lines	qRT-PCR, WB, IHC	T < N
HCC [88]	tissues, cell lines	qRT-PCR, WB, IHC	T < N
HCC [54]	tissues	qRT-PCR, IHC	T < N
HCC [89]	cell lines	qRT-PCR, WB, IHC	T < N
HCC [90]	tissues	qRT-PCR, WB, IHC	T < N
ccRCC [91–93]	tissues	ICGC database, qRT-PCR, IHC	T > N
Breast cancer [39, 94]	tissues	Oncomine database	T > N
NSCLC [95]	tissues	qRT-PCR, IHC	T > N
Lung cancer [94]	tissues	Oncomine database	T < N
Prostate cancer [96]	tissues	GEO database	T > N
Glioma [97]	tissues (human, mouse)	IHC	T > N
Ovarian cancer [74]	tissues	WB	T > N
Colorectal cancer [94]	tissues	Oncomine database	T > N
Colon cancer [94]	tissues	Oncomine database	T > N
Gastric cancer [94]	tissues	Oncomine database	T > N
Astrocytic tumor [98]	tissues	RT-PCR, WB	T > N
Laryngeal cancer [99]	tissues, cell lines	qRT-PCR, IHC	T < N

**Table 5:** AQP9 expression and prognosis of cancer

Cancer types	Expression types	Expression and prognosis
HCC [54, 87]	mRNA, protein	Higher expression shows better OS
Breast cancer [94]	mRNA	Higher expression shows worse OS, RFS
Breast cancer [39]	mRNA	Higher expression shows worse RFS
ccRCC [91, 93]	mRNA, protein	Higher expression shows worse OS
ccRCC [92]	mRNA, protein	Higher expression shows worse OS, PFS
Colorectal cancer [94]	mRNA	Higher expression shows worse DFS
NSCLC [95]	mRNA, protein	Higher expression shows worse OS, DFS
Colon cancer [94]	mRNA	Higher expression shows worse DFS
Gastric cancer [94]	mRNA	Higher expression shows worse OS, PFS
Lung cancer [94]	mRNA	Higher expression shows worse OS, PFS
Laryngeal cancer [99]	mRNA	Higher expression shows worse OS

## Functions in cancer

### AQP3

AQP3 is multifaceted in cancer. As a member of the AQGP family, it is universally acknowledged that AQP3 works as a channel for water and glycerol. Impaired glycerol transport and lipid synthesis due to AQP3 knockdown promoted apoptosis and inhibited the proliferation of gastric cancer cells [13, 14], AQP3-facilitated glycerol, a major source of ATP, participates in epidermal proliferation and tumor formation [15].

**Fig. 2:**
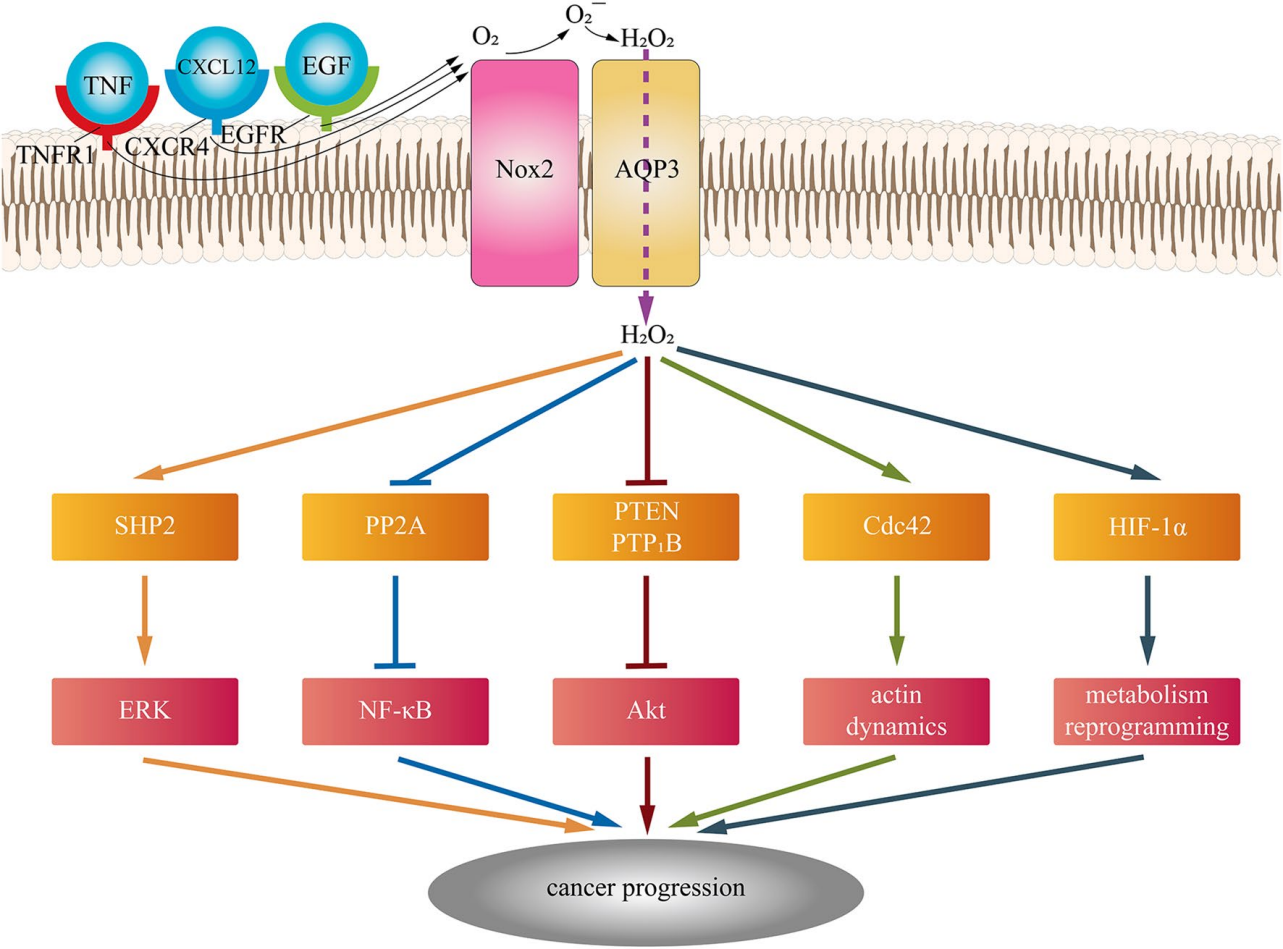
AQP3-mediated H_2_O_2_ promotes cancer progression via tumor-related signaling pathways

In addition to transporting water and glycerol, it can also transport H_2_O_2_, an important second messenger in cellular activities [100], which makes the role of AQP3 in cancer more significant. Extracellular H_2_O_2_, synthesized by NADPH oxidase 2 (Nox2), which responds to various stimuli, including TNF-α, EGF, and CXCL12, is delivered intracellularly through AQP3, and then, H_2_O_2_ inactivates protein phosphatase 2A (PP2A) followed by the regulation of IKKβ and NF-κB/p65 [101]. AQP3-mediated H_2_O_2_ oxidized PTEN and protein tyrosine phosphatase 1B (PTP1B) and activated the Akt pathway in breast cancer cells and lung adenocarcinoma cells [102, 103]. Moreover, AQP3-facilitated H_2_O_2_ engaged in Cdc42 activation, a GTPase of the Rho family and subsequent actin dynamics [104]. In addition, AQP3 was involved in the EGF-induced ERK pathway in cancer, in which AQP3-mediated H_2_O_2_ modulated SHP2, an indispensable part of the downstream MAPK signaling cascade [105, 106]. Moreover, HIF-1α could be upregulated by ROS transported by AQP3, which made a difference in reprogramming cancer metabolism [107, 108]. Figure 2 shows AQP3-mediated H_2_O_2_ in cancer.

AQP3 functions as a functional protein molecule, and knockdown of AQP3 inhibits cancer cell proliferation, invasion, and migration as well as promotes apoptosis [16, 55, 64, 109].

**Fig. 3:**
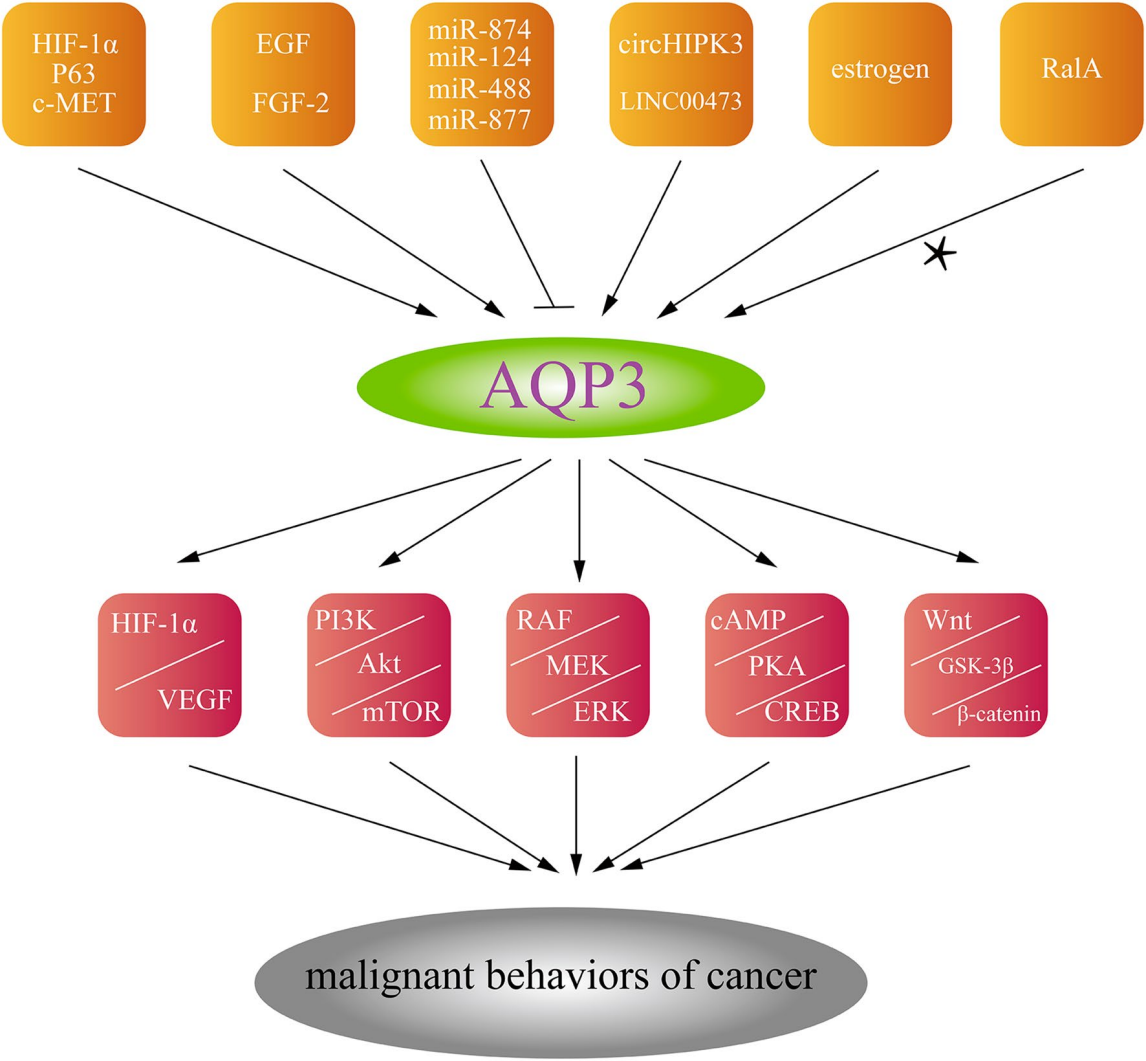
The upstream regulators of AQP3 and the downstream activated tumor-related signaling pathways [13, 49–53, 56, 58–61, 107, 110–124]

There are many transcription factors, cytokines, microRNAs, and other regulators that affect AQP3 in cancer. Likewise, AQP3 can regulate the malignant behaviors of cancer cells through several signaling pathways. Here, we summarize the upstream regulators and the downstream activated tumor-related signaling pathways of AQP3 in different cancers, hoping to provide some basis for AQP3 as a target for cancer treatment (see Fig. 3, for more details).

Growing evidence shows that some metal compounds modulating the expression of AQP3 exhibit different anticancer properties, such as antiproliferative and proapoptotic properties. In vivo, Auphen could regulate the expression of AQP3 to inhibit tumor growth and promote apoptosis [54]. P2W18, a polyoxotungstate, showed the ability to suppress cancer cell migration mainly by affecting AQP3, implying the potential of AQP3 as an anticancer agent in tumors with high AQP3 expression [125]. Some natural compounds also show anticancer ability to some degree. Curcumin, which regulates AQP3 gating [126], exerted an inhibitory effect on EGF-induced AQP3 upregulation and ovarian cancer cell migration through the PI3K/Akt and MEK/ERK pathways [127]. Similarly, Manuka honey accelerated epithelial cancer cell apoptosis by maintaining the high permeability of AQP3-induced H_2_O_2_ [128].

AQP3 can interact with certain chemotherapy drugs or participate in certain cancer treatments. AQP3 gave rise to chemoresistance to cisplatin in gastric cancer and facilitated chemoresistance to arsenite in melanoma [129, 130]. Meanwhile, AQP3 also participates in the cytotoxic effect exerted by nucleoside-derived drugs, including 5-fluorouracil and gemcitabine, in breast cancer and colon cancer [131]. Cryotherapy is gradually becoming an alternative treatment for the early stage of the neoplastic process, in which AQP3 plays a role in gilding. Breast cancer cells and prostate cancer cells treated with AQP3-siRNA were more sensitive to cryoinjury than control-siRNA [132, 133]. Thus, inhibition of AQP3 may be a potential adjunct to cryotherapy for breast and prostate cancer patients.

### AQP7 and AQP10

AQP7 is regarded as a gateway for water and glycerol transportation, but little work has been performed on its involvement in tumor cell lipid metabolism. Nevertheless, AQP7, which serves as an important target for arsenite uptake in mammals [134], may provide us with novel perceptions of its chemotherapeutic efficacy in acute promyelocytic leukocytes. AQP7 regulated multiple metabolic pathways, including lipid metabolism, urea metabolism, and carbohydrate metabolism and activated p38, EGFR, and mTOR signaling cascades. In addition, AQP7 made cells more sensitive to the oxidative environment [17]. In other words, AQP7, as a critical regulator, might eventually lead to the development of more effective therapeutics in breast cancer.

Studies have shown that silencing AQP7 in adipose cells could increase the glycerol content, strengthen the activity of the Gyk enzyme, and promote the accumulation of triglycerides [135]. When the body needs energy, triglycerides are hydrolyzed into free fatty acids (FFAs) and glycerol, glycerol is delivered to the liver to participate in gluconeogenesis, FFAs are transported to mitochondria where energy is produced, and AQP7 functions as the glycerol gateway during the process [136]. Another study demonstrated low glycerol and ATP contents in the hearts of KO-AQP7 mice [137]. Therefore, we infer that low expression of AQP7, which leads to an increased content of triglycerides, impaired glycerol and FFA transport, and reduced energy, inhibits the malignant behaviors of tumor cells. Moreover, the role of AQP10 in cancer has never been satisfactorily elucidated, which means that more research regarding AQP7 and AQP10 in cancer is needed.

### AQP9

The involvement of AQP9 in glycerol transportation continues to draw attention from researchers, and now it has been extended to cancer research. In several cancer cell lines, the expression of AQP9 was related to the uptake of [14C]-labeled glycerol [138]. Another experiment in a rat hepatocellular carcinoma model found that the expression of AQP9 was present at a low level before tumorigenesis, while it was significantly increased in the early stage of hepatocellular carcinoma. This indicates a transition of glycerol metabolism during the stage [18].

Our team found that AQP9 plays an extraordinary role in the prostate. First, we proved the positive regulatory effect of androgen on AQP9 in the prostate in vitro and in vivo [139]. In addition, knockdown of AQP9 inhibited proliferation, migration, and invasion as well as promoted apoptosis in androgen-independent prostate cancer, which is involved in the ERK pathway [96]. We can conclude that AQ9 accelerates prostate cancer progression in combination with the relatively high expression level of AQP9 in prostate cancer tissues compared with normal prostate tissues. Specific targeted therapy with AQP9 might exert far-reaching significance in prostate cancer treatment.

In renal cell carcinoma, Yasutaka Yamada et al. found that AQP9 was regulated by miR-532, silencing AQP9 could affect the oncological behaviors of renal cancer cells [91], and a cancer-promoting effect via the Akt pathway was also found in astrocytoma [140].

**Fig. 4:**
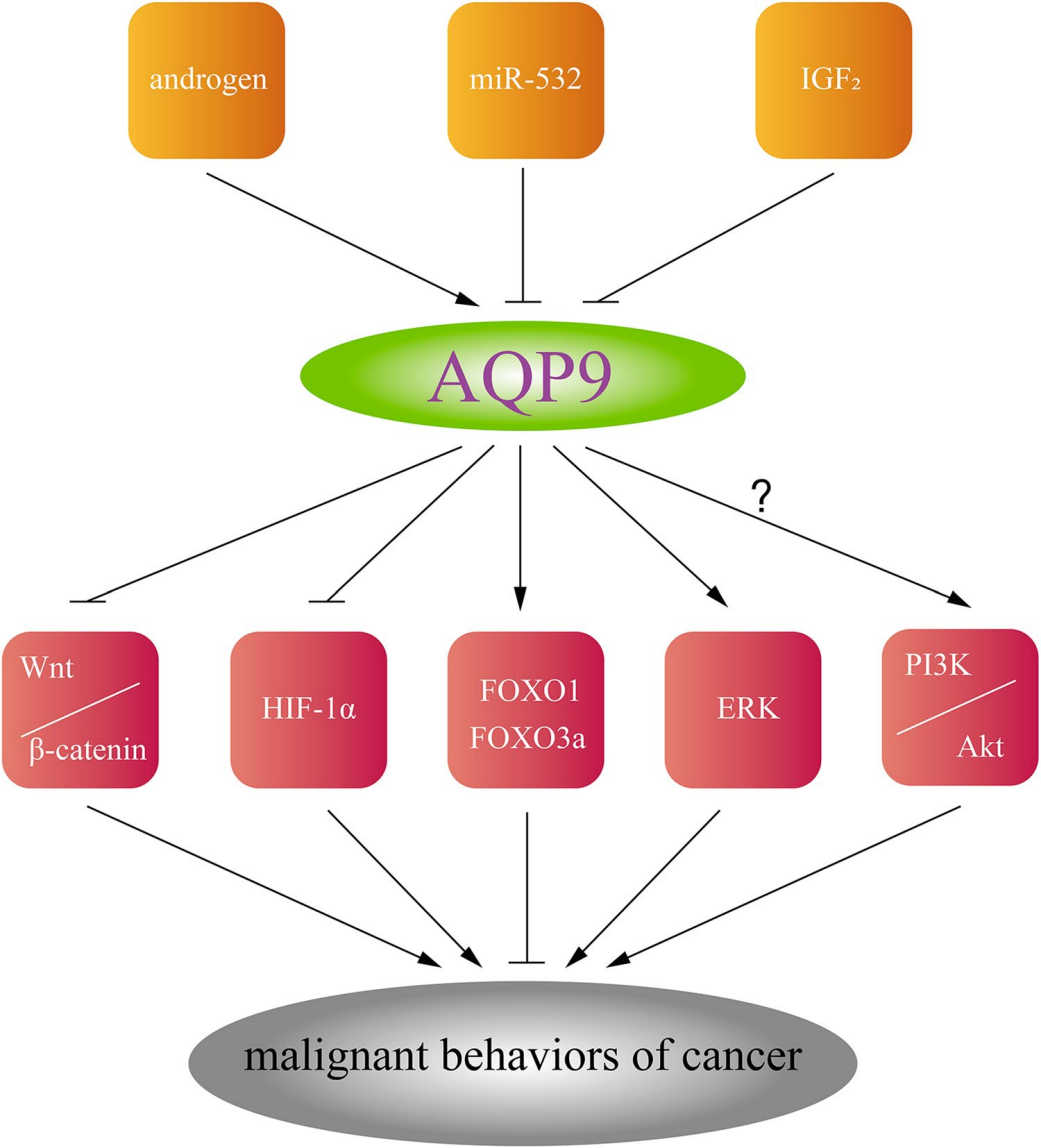
The upstream regulators of AQP9 and the downstream activated tumor-related signaling pathways

For hepatocellular carcinoma, AQP9 suppresses hepatocellular carcinoma cell growth and metastasis via distinct pathways, including HIF-1α, PI3k/Akt, Wnt/β-catenin, and FOXO1 [86–88, 90, 141]. Further findings from another study announced the role of AQP9 in H_2_O_2_ transport, as Sachiko Watanabe et al. reported [142]. In this study, the author proved that AQP9 mediated by insulin-like growth factor 2 (IGF2), inhibited liver cancer stem cell stemness through ROS/β-catenin/FOXO3a [143]. Figure 4 shows the upstream regulators of AQP9 and the downstream activated tumor-related signaling pathways.

With respect to additional functions of AQP9, it regulated arsenic transportation and affected As2O3 sensitivity [134, 144, 145]. The expression level of AQP9 was related to sensitivity to As2O3 in acute promyelocytic leukemia [146], and azacytidine upregulated AQP9 to make acute myeloid leukemia cells more sensitive to As2O3 [147].

AQP9 is also involved in the chemotherapy effects of a variety of solid tumors. AQP9 enhanced the chemotherapy response and alleviated the chemotherapy resistance of arsenic during the treatment of lung cancer [148, 149]. In contrast, AQP9 fostered the chemotherapy resistance of melanoma to arsenite [129]. 5-FU chemotherapy possesses a better curative effect in mice with colorectal cancer because of cell cycle arrest caused by AQP9 [150]. The expression of AQP9 at a low level in patients with stage III colorectal cancer who do not respond to chemotherapy, makes AQP9 a potential prognostic indicator [151]. Moreover, the functions of AQP9 in glycerol transportation and differential expression between tumors and normal tissues make AQP9 a promising scientific hot button for the treatment of various tumors.

## Conclusions and perspectives

During the past decade, great achievements have been witnessed in the research of aquaporins, from the location of genetic information, distribution and function to the transport mechanism, drug mechanism, etc. Aquaglyceroporins, as a special group from the aquaporin family, have been rooted in researchers’ minds.

AQP3 and AQP9, which are permeable to glycerol and H_2_O_2_, often contribute to the malignant behaviors of cancer. However, AQP3 plays an opposite role in endometrioid carcinoma and MIBC, as well as AQP9 in hepatocellular carcinoma. AQP7 is involved in multiple metabolic pathways in breast cancer while its functions in other cancers remain to be explored. In addition, they all facilitate arsenic transportation or affect its chemotherapy effect, making them hopeful therapeutic targets in cancer treatments. However, further analysis at the protein level is needed, especially for AQP7 and AQP10.

This review shows that AQGPs behave as double-edged swords in different tumors. They have different distributions and expression patterns from each other in cancers, and they are linked to different oncological behaviors of tumor cells, including proliferation, migration, invasion, apoptosis, epithelial–mesenchymal transition, metastasis, etc.

To the best of our knowledge, this is the first review on aquaglyceroporins that combines clinical and basic research. We calculated the relationship between the expression levels of AQGPs and prognostic indicators in different tumors in the published literature. We also summarized the upstream and downstream regulators and signaling pathways involved, which may provide some references for subsequent further research on AQGPs and drug treatments specifically targeting AQGPs. Despite these findings, we still cannot provide a systematic and complete explanation of the mechanism of AQGPs in cancer; further basic researches about what role AQPs may play and how they regulate the reprogramming of lipid metabolism in tumor cells are needed.
